# A cross-sectional feasibility study of neurovascular ultrasound in Malawian adults with acute stroke-like syndrome

**DOI:** 10.1371/journal.pone.0229033

**Published:** 2020-02-07

**Authors:** Joseph Kamtchum-Tatuene, Henry C. Mwandumba, Gloria Mwangalika Kachingwe, Laura J. Bonnett, Noel Kayange, Tom Solomon, Laura A. Benjamin

**Affiliations:** 1 Malawi-Liverpool-Wellcome Trust Clinical Research Programme, University of Malawi College of Medicine, Blantyre, Malawi; 2 Department of Clinical Sciences, Liverpool School of Tropical Medicine, Liverpool, England, United Kingdom; 3 Department of Biostatistics, University of Liverpool, Liverpool, England, United Kingdom; 4 Department of Medicine, College of Medicine, University of Malawi, Blantyre, Malawi; 5 Institute of Infection and Global Health, University of Liverpool, Liverpool, England, United Kingdom; Columbia University Medical Center, UNITED STATES

## Abstract

**Background:**

In sub-Saharan Africa, there is a dearth of epidemiologic data on the burden of cerebral atherosclerosis. This is explained by the limited availability and the high cost of standard vascular imaging techniques. Neurovascular ultrasound is portable, cheaper and non-invasive and could, therefore, represent a reasonable alternative to fill this knowledge gap. We explored the feasibility of neurovascular ultrasound in Malawian adults with acute stroke-like syndrome to inform the design of future large stroke studies comparing its diagnostic performance to that of gold standard vascular imaging techniques in sub-Saharan Africa.

**Methods:**

We enrolled consecutive patients diagnosed with acute stroke-like syndrome based on the World Health Organization definition. Clinical and demographic data were recorded, and a comprehensive neurovascular ultrasound was performed. Fisher’s exact and Kruskal-Wallis tests were used to study the relationship between atherosclerosis and potential risk factors.

**Results:**

Sixty-six patients were enrolled (mean age: 58.7 years). The frequency of extracranial atherosclerosis was 39.4% (n = 26, 95% CI: 28.6–52.2). There were 12 patients with abnormal carotid intima media thickness (18.2%, 95% CI: 9.8–29.6) and 14 patients with a carotid plaque (21.2%, 95% CI: 12.1–33.0). The frequency of intracranial atherosclerosis was 19.2% (95%CI: 6.6–39.4) in 26 patients with successful transcranial insonation. Hypertension (80.8 versus 52.5%, p = 0.03) and hypercholesterolemia (11.5 versus 0.0%, p = 0.05) were more prevalent in patients with extracranial atherosclerosis.

**Conclusions:**

This study demonstrates the feasibility of neurovascular ultrasound to assess cervical arteries in adults with stroke-like syndrome in sub-Saharan Africa. There is a high rate of transcranial insonation failure in this setting, highlighting the need for echocontrast agents.

## Introduction

Intracranial atherosclerosis is responsible for 8%-10% of ischemic strokes in the western world and 30%-50% of ischemic strokes in Asia [1, 2]. In sub-Saharan Africa, the contribution of intracranial atherosclerosis to the stroke burden is unknown. In Nigeria, a middle-income country, one study using computed tomography in 130 consecutive patients with ischaemic stroke demonstrated the presence of intracranial arterial calcifications in more than 90% of cases [3]. Intracranial arterial calcifications represent an integral part of the atherosclerotic process though they do not necessarily correspond to clinically significant disease. Another pilot autopsy study in the same country identified intracranial atherosclerosis in 45% of 44 patients but the cause of death and the proportion of stroke cases was not specified [4].

The dearth of epidemiologic data on the burden of intracranial atherosclerosis in Africa could be explained by the limited availability and the high cost of standard vascular imaging tools such as computed tomography, magnetic resonance and digital subtraction angiography. Neurovascular ultrasound examination is portable, cheaper and non-invasive. It could, therefore, represent a reasonable alternative to fill this knowledge gap if proven feasible and reliable. Indeed, the sensitivity and the specificity of neurovascular ultrasound are generally greater than 90% for the diagnosis of cervical and intracranial atherosclerosis [5, 6]. In this preliminary study, we aimed to explore the feasibility of neurovascular ultrasound in Malawian adults with an acute stroke-like syndrome. The data collected would inform the design of future large stroke studies comparing the diagnostic performance of neurovascular ultrasound to that of gold standard vascular imaging techniques in sub-Saharan Africa.

## Methods

### Ethics approval and consent to participate

This work was conducted as part of the study of Biomarkers of Stroke and Arterial diseases in Malawian Adults (BIOSTATA). The latter received ethical clearance from the Research Ethics Committees of the Liverpool School of Tropical Medicine (authorization 16–035) and the University Of Malawi College Of Medicine (authorization P.06/16/1974). A written informed consent was obtained from all the patients enrolled or their next of kin.

### Selection of patients and clinical assessment

Patients with an acute stroke-like syndrome were recruited consecutively from April to August 2017 at the Queen Elizabeth Central Hospital, the largest teaching and referral hospital in Malawi. Stroke-like syndrome was clinically defined as a rapidly developing focal or global neurologic impairment, lasting more than 24 hours, with no apparent cause other than of vascular origin (WHO definition) [7, 8]. Due to budgetary restrictions, the diagnosis of stroke could not be confirmed by brain imaging in this feasibility study as was the case in former better funded studies in the same setting [9].

For each participant enrolled, the following data were recorded: age, sex, hypertension and diabetes status, tobacco and alcohol consumption status, body temperature, blood pressure, body mass index, and waist-hip ratio. Blood samples were collected for random blood sugar, full blood count, and lipid profile. The operational definitions for hypertension, diabetes, and hypercholesterolemia, were reported previously [9]. Hypertension was defined as a blood pressure > 140/90 mmHg or use of antihypertensive medication. Diabetes mellitus was defined as random blood sugar > 11.1 mmol/l or use of glucose-lowering medication. Hypercholesterolemia was defined as non-fasting serum total cholesterol levels > 6.2 mmol/l or use of lipid-lowering medication.

### Ultrasound assessment

To characterise atherosclerosis, all participants underwent a comprehensive neurovascular ultrasound examination performed on the day of enrolment by a certified neurosonologist with 2 years of experience (JK-T), using the CX50 portable ultrasound device (Philips, Amsterdam, NL).

The assessment of cervical extracranial arteries comprised a semi-automated measurement of the carotid intima-media-thickness (cIMT) using the QLAB 10.0 software (Philips, Amsterdam, NL) and a grading of any existing stenosis using the multidisciplinary consensus criteria for the diagnosis of carotid stenosis provided by the Society of Radiologists in Ultrasound (S1 File) [10]. The percentage of the carotid lumen occupied by the visible plaque is one of the two primary parameters used by the Society of Radiologists in Ultrasound for the grading. In this study, it was measured using the method described by the European Carotid Surgery Trial (ECST) [11]. The cIMT was measured on a straight segment of the far wall of the common carotid artery (CCA), between 10 and 30 mm below the bifurcation. Two values were obtained on each CCA. The Mannheim consensus recommendations were applied to identify patients with abnormal cIMT using the average of the two values obtained on each CCA [12]. In order to account for the normal aging of the vessel wall, the Mannheim consensus guidelines suggest using age-adjusted cut-offs to define abnormal cIMT. In this study, the following cut-offs were applied: 0.60 mm (30–49 years old), 0.70 mm (50–59 years old), 0.75 mm (60–69 years old), and 0.80 mm (≥70 years old) [12].

The assessment of intracranial arteries was performed using transcranial color-coded duplex sonography (TCCS). The insonation was done through the temporal and occipital windows. The peak systolic velocity (PSV) and mean flow velocity (MFV) were recorded on several portions of the large intracranial arteries: middle, anterior, and posterior cerebral arteries; intracranial segments of the vertebral arteries also known as V4; carotid siphons and the basilar artery. These values were then used for the diagnosis and grading of intracranial stenosis (S1 File). The primary criteria were represented by abnormally high values of the MFV and asymmetry between the right and the left for each arterial segment. When the decision was not clear, secondary criteria were applied, notably high values of PSV and the stenotic/prestenotic ratio (SPR) of mean flow velocities [13].The SPR is the ratio of the MFV in the abnormal/stenotic arterial segment to the MFV in the closest normal upstream segment. The quality of the insonation window was graded as good, poor, or absent. A good insonation window would allow the visualization of the entire course of the intracranial arteries and the measurement of blood flow velocities using the standard technical settings of the ultrasound device. A poor insonation window would only allow partial visualization of the intracranial arteries (only small independent segments of the arteries are visible) with reduced image quality and the measurement of blood flow velocities, if possible, would require adjusting the technical settings (decreasing the size of the insonation gate, increasing the signal gain). When the insonation window is absent, it is impossible to visualize the intracranial arteries or measure the blood flow velocities. Contrast enhanced TCCS and power mode Doppler were not available.

### Operational definitions

Depending on the presence, the topography (extracranial or intracranial) and the severity (degree of stenosis) of the atherosclerosis identified on the carotid-vertebral and transcranial ultrasound examinations, we defined:

*Intracranial atherosclerosis* as the presence of any abnormal finding on TCCS.

*Extracranial atherosclerosis* as the presence of any abnormal finding on carotid-vertebral ultrasound and, therefore, encompassing abnormal carotid intima-media thickness and extracranial carotid or vertebral plaques.

*Abnormal carotid intima-media thickness* as thickening of the intima-media layer of the carotid wall with no plaque on carotid-vertebral ultrasound. This represents the early stage of carotid atherosclerosis. The diagnosis was made if the patient had an abnormal value of cIMT on at least one CCA (right or left, cut-offs described above). The two CCA were considered individually to maximize the sensitivity, considering that atherosclerosis is not a uniform symmetrical process.

*Plaque* as the presence of a focal structure protruding into the lumen that is at least 50% or 0.5 mm thicker than the surrounding IMT or a focal structure encroaching into the lumen and demonstrating an overall thickness of > 1.5 mm as measured from the intima-lumen interface to the media-adventitia interface on B-mode carotid-vertebral ultrasound [12].

A carotid, vertebral or intracranial stenosis was considered *clinically significant* if ≥ 50%.

### Statistical analysis

The statistical analyses were performed using the software STATA (version 13, StataCorp, College Station, TX, USA). Participants’ demographic and clinical parameters were summarized as proportions and means with 95% confidence interval for categorical and numerical variables, respectively.

We explored the relationship between the atherosclerosis subtypes and the following factors: age, sex, hypertension, diabetes mellitus, tobacco and alcohol consumption, waist-hip ratio, and hypercholesterolemia. The Fisher’s exact test was used for categorical variables and the Kruskal-Wallis test for continuous variables.

All the statistical tests performed were two-tailed and statistical significance was defined as p ≤ 0.05.

## Results

### Participants’ characteristics

**Fig 1:**
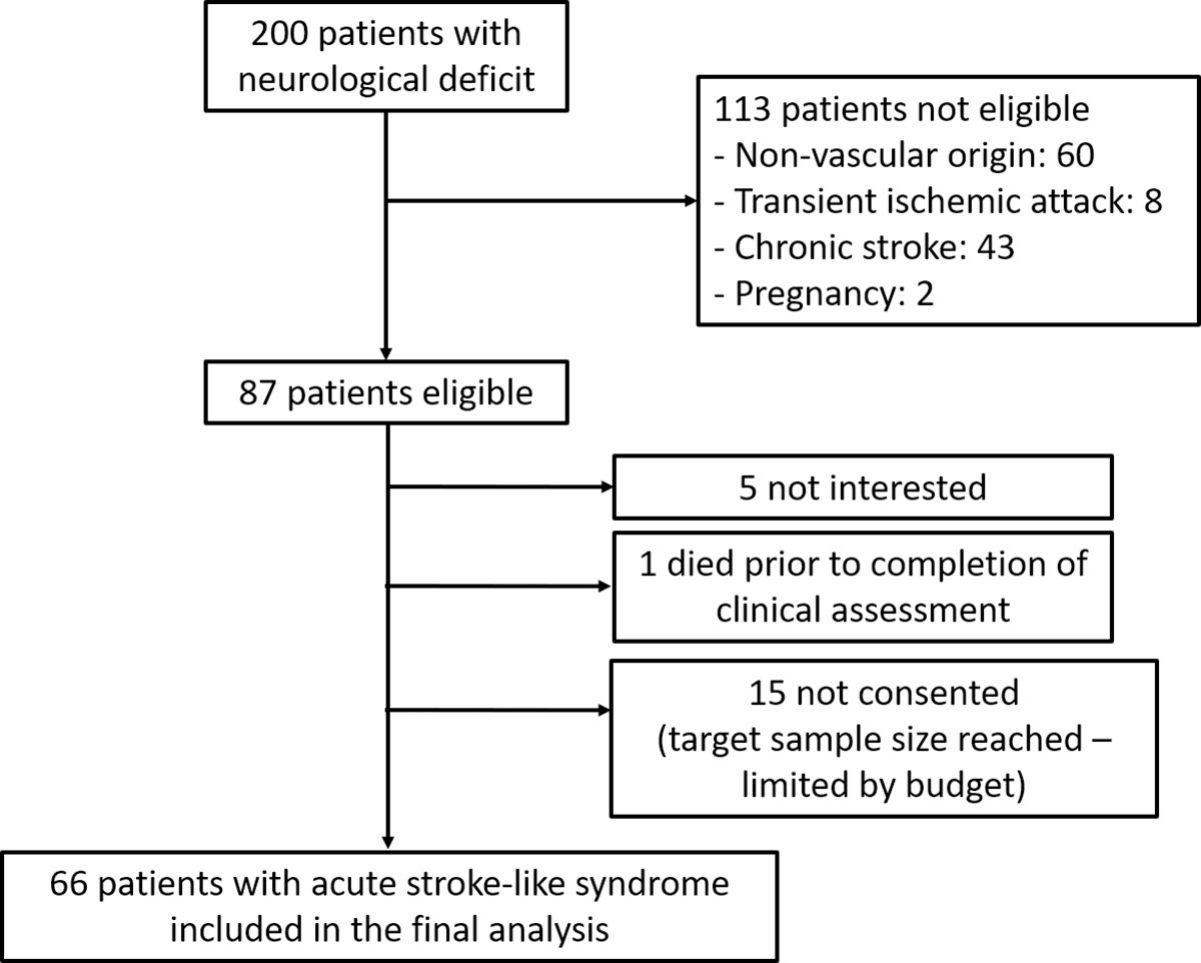
Overview of the patients’ selection process.

Overall, 200 patients admitted with a neurologic deficit were screened and 66 patients with an acute stroke-like syndrome were included (Fig 1). The mean age was 58.7 years (95% CI: 54.4–63.1). Participants’ baseline characteristics are summarized in Table 1.

**Table 1:** Baseline characteristics of patients presenting with an acute a stroke-like syndrome.

Characteristics	N (%) or mean (95%CI)
**Age (years)**	58.7 (54.4–63.1)
**Men**	28 (42.4)
**Onset-to-enrolment time (days)**	3.2 (2.8–3.6)
**Smoking**	8 (12.1)
**Alcohol cons.**	13 (19.7)
**Hypercholesterolemia**	3 (4.6)
**Hypertension**	42 (63.6)
**Diabetes**	11 (16.7)
Waist-hip ratio*	0.90 (0.83–0.94)

* Median with interquartile range

### Frequency of intracranial atherosclerosis

The temporal windows were rated as good, poor, or absent in 20, 10, and 36 patients on the right versus 17, 11, and 38 patients on the left. Both temporal windows were absent in 34 patients. The occipital window was rated as good, poor, or absent in 41, 20, and 5 patients. All patients with a missing occipital window also had both temporal windows missing. In the absence of other vascular imaging modalities, asymmetry of blood flow velocities was a primary criterion for the diagnosis of intracranial atherosclerosis. Therefore, we could not reliably interpret the TCCS findings in patients with one missing temporal window. Forty patients had at least one absent insonation window on TCCS. The TCCS findings are presented for the remaining 26 patients.

Intracranial atherosclerosis was found in 5 patients (19.2%, 95% CI: 6.6–39.4) and three of them also had extracranial atherosclerosis (2 with carotid plaque and 1 with abnormal carotid intima-media thickness). The mean age was 55.2 years (95% CI: 23.0–87.4). The topography and the severity of the intracranial atherosclerosis are summarized in Table 2.

**Table 2:** Topography and severity of the intracranial atherosclerosis diagnosed with transcranial color-coded duplex sonography^a^.

ID	Age (years)	Anterior circulation						Posterior circulation					Number of vessels affected
		Right			Left			Right		Left		BA	
		MCA	ACA	Siphon	MCA	ACA	Siphon	PCA	V4	PCA	V4		
**1**	70	No	No	No	No	No	No	Yes (<50%)	No	No	No	No	01
**2**	75	No	No	No	Yes (>70%)	No	No	Yes (<50%)	Yes (50–70%)	No	No	No	03
**3**	76	Yes (50–70%)	No	No	No	No	No	Yes (50–70%)	No	Yes (50–70%)	No	No	03
**4**	35	No	No	No	Yes (>70%)	No	No	No	No	No	No	No	01
**5**	20	No	No	No	Yes (>70%)	No	No	No	No	No	No	No	01

^a^ When there is an intracranial atherosclerosis, the degree of stenosis is indicated in brackets

MCA = Middle Cerebral Artery, ICA = Internal Carotid Artery, PCA = Posterior Cerebral Artery, ACA = Anterior Cerebral Artery, BA = Basilar Artery, VA = Vertebral Artery (this refers only to the intracranial portion)

### Frequency of extracranial atherosclerosis

The frequency of extracranial atherosclerosis was 39.4% (n = 26, 95% CI: 28.6–52.2). The mean age was 63.0 years (95% CI: 57.7–68.2).

Abnormal carotid intima-media thickness was found in 12 patients (18.2%, 95% CI: 9.8–29.6). Their mean age was 61.8 years (95% CI: 52.6–70.9).

A carotid plaque was found in 14 patients (21.2%, 95%CI: 12.1–33.0) including 5 with normal TCCS, 2 with intracranial atherosclerosis, and 7 with unsuccessful TCCS. No plaque was identified on the vertebral arteries.

**Fig 2:**
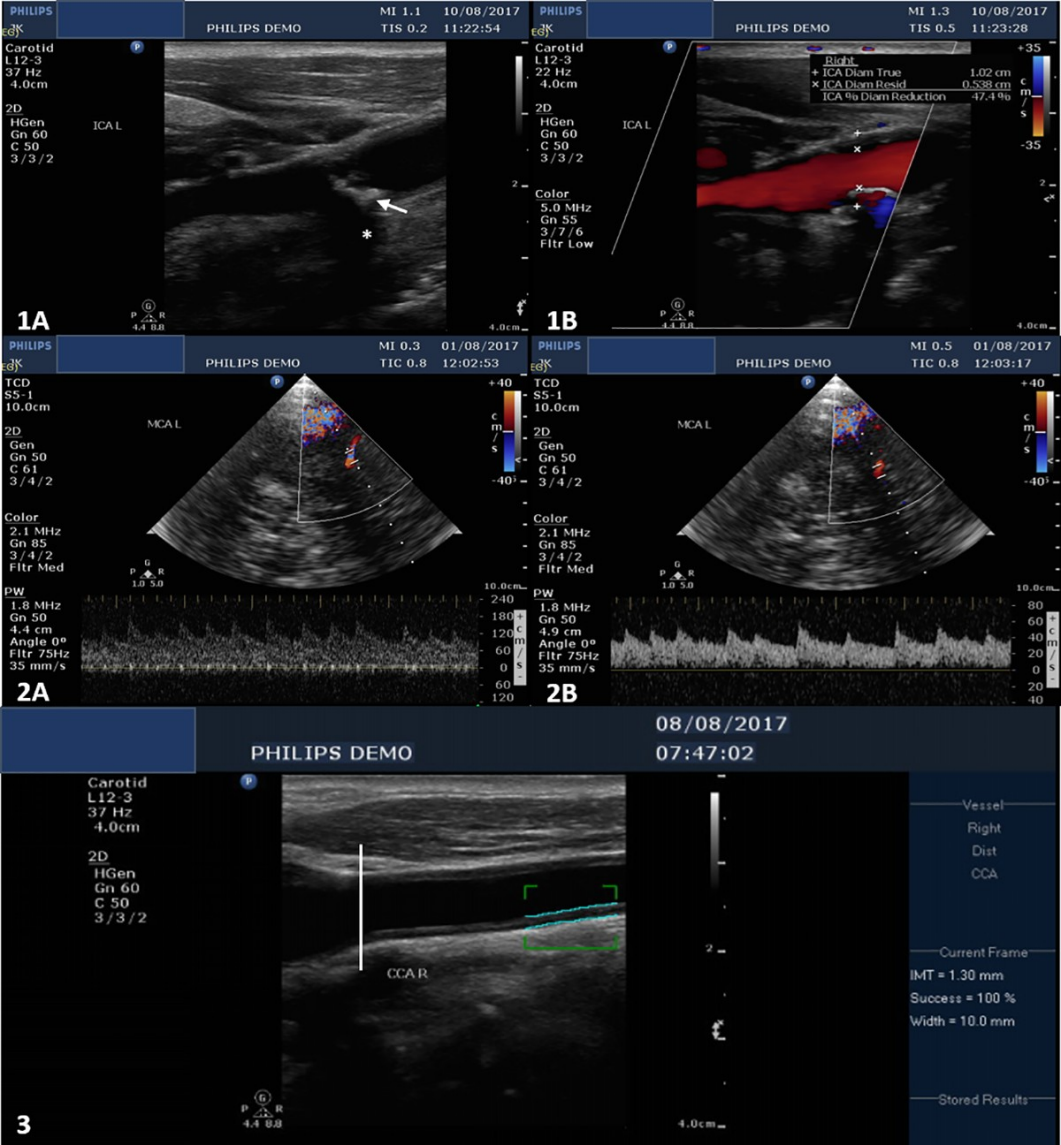
Illustrative cases of extracranial and intracranial atherosclerosis. Case 1: Left carotid artery stenosis (B mode carotid ultrasound–longitudinal section). There is an isoechoic regular plaque (1A –white arrow) of the left carotid bulb. The partial calcification of the plaque creates an acoustic shadow (1A –white asterisk). The plaque causes a 47% diameter reduction as illustrated on the colour-coded duplex image (1B). Case 2: Left middle cerebral artery stenosis of more than 70% (colour-coded duplex and spectra). 2A: Stenotic segment (44 cm depth): PSV = 187 cm/s, MFV = 130 cm/s. 2B: Prestenotic segment (49 cm depth): PSV = 62 cm/s, MFV = 40 cm/s. The stenotic/prestenotic ratio is 3.25. PSV = peak systolic velocity, MFV = mean flow velocity.Case 3: Abnormal intima-media thickening (B mode carotid ultrasound–longitudinal section). The carotid intima-media thickness is measured between 10 and 30 mmm below the carotid bifurcation (white vertical line).

None of the participants had a clinically significant carotid stenosis. The highest degree of carotid stenosis identified was 47% (Fig 2).

### Risk factors of atherosclerosis

All patients with intracranial atherosclerosis were males. There was no association between intracranial atherosclerosis and hypertension, diabetes mellitus, hypercholesterolemia, smoking, alcohol consumption, and waist-hip ratio. Hypertension and hypercholesterolemia were associated with the presence of extracranial atherosclerosis (Table 3).

**Table 3:** Exploratory univariable analysis of the factors associated with intracranial and extracranial atherosclerosis.

Characteristic	Intracranial atherosclerosis			Extracranial atherosclerosis		
	Yes (n = 5)	No (n = 21)	p	Yes (n = 26)	No (n = 40)	p
**Age (years)**	55.2 (23.0–87.4)	52.9 (45.7–60.1)	0.80	63.0 (57.7–68.2)	56.1 (49.7–62.4)	0.12
**Men**	5 (100.0)	9 (42.9)	0.03	13 (50.0)	15 (37.5)	0.44
**Smoking**	1 (20.0)	1 (4.8)	0.35	3 (11.5)	5 (12.5)	1.0
**Alcohol consumption**	1 (20.0)	1 (4.8)	0.35	4 (15.4)	9 (22.5)	0.54
**Hypercholesterolemia**	0 (0.0)	1 (4.8)	1.00	3 (11.5)	0 (0.0)	0.05
**Hypertension**	3 (60.0)	10 (47.6)	1.00	21 (80.8)	21 (52.5)	0.03
**Diabetes mellitus**	0 (0.0)	3 (14.3)	1.00	5 (19.2)	6 (15.0)	0.74
**Waist-hip ratio**	0.9 (0.8–1.0)	0.9 (0.8–1.0)	0.49	0.9 (0.9–1.0)	0.9 (0.8–0.9)	0.26

## Discussion

This study was conducted to explore the feasibility of neurovascular ultrasound in Malawian adults with a stroke-like syndrome. Over a period of 5 months, 66 participants were enrolled and non-contrast TCCS was successfully performed in 39.4%. Intracranial atherosclerosis was identified in 5 patients (19.2%). Abnormal carotid intima media thickness and carotid plaques were found in 18.2% and 21.2% of the participants, respectively. Hypertension and hypercholesterolemia were risk factors for extracranial atherosclerosis.

On one hand, this study demonstrates that, carotid-vertebral ultrasound may be used as an alternative to conventional cerebrovascular imaging techniques that are more invasive, more expensive, and less widely available in sub-Saharan Africa. Further studies are warranted for defining age- and sex-adjusted reference values of flow velocities and cIMT and clarifying its sensitivity and specificity for the diagnosis of atherosclerosis in sub-Saharan Africa. On the other hand, we report a high failure rate of TCCS, confirming findings from previous studies indicating that people of African descent, females and elderly subjects have higher rates of transcranial insonation failure due to greater thickness of the temporal bone [14]. This suggests that using echocontrast agents might be mandatory for the study of intracranial atherosclerosis in African settings when other imaging techniques are not affordable. Indeed, the adjunction of echocontrast agents converts 65 to 80% of unsuccessful transcranial ultrasound examinations into diagnostic investigations [15–17]. Despite their relatively high cost, echocontrast agents may be easier to afford financially and logistically through subsidized health programs and international research collaborations than the acquisition and deployment of advanced vascular imaging devices.

The limited epidemiologic data available in Africa show a relatively low prevalence of extracranial atherosclerosis and a high proportion of cryptogenic strokes [18]. It is, therefore, assumed that a large proportion of strokes might be related to undiagnosed intracranial atherosclerosis as seen in high income countries. In our cohort, intracranial atherosclerosis was diagnosed in 1 out of 5 patients with a successful TCCS. This relatively high frequency of intracranial atherosclerosis might be related to the high prevalence of hypertension and to the effects of the rampant epidemiological transition in Malawi with rapidly increasing prevalence of other cardiovascular risk factors such as diabetes mellitus, hypercholesterolemia, obesity, and smoking [19]. Indeed diabetes mellitus, dyslipidaemia and tobacco consumption are major contributors to the pathophysiology of intracranial atherosclerosis [20]. The lack of association between intracranial atherosclerosis and cardiovascular risk factors in this study is an atypical result likely explained by the small sample size and the low success rate of TCCS.

The frequency of carotid plaque was 21.2% which is similar to the 16.4% prevalence of large artery disease reported in a former stroke study in Malawi that did not include abnormal carotid intima-media thickness [18]. Other studies in Africa have reported a similar prevalence of carotid plaque [21]. Despite the presence of a carotid plaque, none of our patients had a degree of stenosis above the threshold of clinical significance (>50%) that could support an association with their stroke. Like other African countries [19], as Malawi progresses through the epidemiological transition the prevalence and the severity of both intracranial and extracranial atherosclerosis may rise, thus contributing to a greater burden of stroke in the future. It is therefore important to intensify public health campaigns aimed at improving the diagnosis and the control of major cardiovascular risk factors in the population.

The major strength of this study is the comprehensive and rigorous assessment of both extra- and intracranial cerebral arteries in all participants and the extensive recording of information related to potential risk factors of cerebral atherosclerosis. Moreover, the results are not influenced by the presence of calcifications of the vessel wall that frequently lead to an overestimation of the burden of intracranial atherosclerosis in CT-based studies [3]. However, there are some limitations. First, the ultrasound findings were not confirmed by a more sensitive vascular imaging modality. Although TCCS is validated for the diagnosis and monitoring of intracranial atherosclerosis, it can underestimate the overall burden of intracranial atherosclerosis. Indeed, its sensitivity is decreased in case of poor-quality insonation windows [14] and in mild stenoses without hemodynamic repercussions. Moreover, the examination of the extracranial vertebral artery is technically challenging, the portions outside the vertebral canal are often difficult to identify with confidence and not always accessible. Therefore, diseases preferentially affecting those portions may be missed (V0 or ostial stenosis, and V3 dissection). Additionally, hemodynamic changes due to normal anatomical variations are frequently mislabelled as pathologic. TCCS is also unable to assess the more distal branches of large intracranial arteries as well as small perforating blood vessels. Nevertheless, it should be noted that, based on previous studies [18], a significant prevalence of brain small vessel disease was not expected. The second limitation of our work is the unavailability of routine brain imaging for patients presenting with sudden onset neurological deficits in Malawi. This limited our ability to type the stroke and exclude mimics. The inclusion of mimics might have resulted in an underestimation of the frequency of intracranial atherosclerosis. However, former studies in the same setting have demonstrated that, when applied by an experienced clinician, the WHO clinical definition of stroke is accurate in almost 85% of cases [9]. So, the absence of brain imaging is unlikely to have had a significant impact on our assessment of the burden of cerebral atherosclerosis in the population studied. Furthermore, similarities in the proportion of hypertension, diabetes, hypercholesterolemia, and carotid plaques, from a study of MRI-confirmed strokes in Malawi, provides some internal validation [18].

## Conclusions

Our study demonstrates that using neurovascular ultrasound to assess cervical and intracranial arteries in patients with suspected stroke is feasible though contrast imaging might be needed in the majority of cases to achieve successful transcranial insonation. It also confirms the high prevalence of cardiovascular risk factors potentially contributing to the high frequency of cervical and intracranial atherosclerosis. Further studies on a larger sample, with a more comprehensive work-up of stroke cases, are warranted to validate these results and clarify the pathophysiology of atherosclerosis and stroke in African populations.

## Supporting information

S1 File Classification of cerebral atherosclerosis using findings on cervical and transcranial ultrasound examination. (PDF)

S2 File Study dataset. (DTA)
