# Supplementary material for: A cross-sectional feasibility study of neurovascular ultrasound in Malawian adults with acute stroke-like syndrome
Source: PLoS One. 2020 Feb 7;15(2):e0229033. doi: 10.1371/journal.pone.0229033 (PMC7006928; doi:10.1371/journal.pone.0229033)
Supplement: S1 File — (PDF) [file pone.0229033.s001.pdf]

Classification of cerebral atherosclerosis using findings on cervical and transcranial ultrasound examination

S1 Table: Society of Radiologists in Ultrasound (SRU) consensus criteria for the diagnosis of internal carotid artery stenosis

The multidisciplinary consensus criteria provided by the Society of Radiologists in Ultrasound were used for the identification and grading of carotid stenosis [1]. These criteria combine flow velocity values and visual estimates of the internal carotid artery (ICA) diameter reduction. This diameter reduction was measured using the European Carotid Surgery Trial (ECST) method which has several advantages including good reproducibility between experienced observers, estimation of the stenosis closer to area values, widespread use and firm prognostic data regarding the risk of stroke and benefit of CEA [2].

**Stenosis grade (%)**	**Primary parameters**		**Secondary parameters**	
	**ICA PSV (cm/s)**	**Diameter reduction (%) using ECST method**	**ICA/CCA PSV ratio**	**ICA EDV (cm/s)**
**Normal**	< 125	None	< 2.0	< 40
**< 50**	< 125	< 50	< 2.0	< 40
**50 – 69**	125 – 230	≥ 50	2.0 – 4.0	40 – 100
**≥ 70 but less than near occlusion**	> 230	≥ 50	> 4.0	> 100
**Near occlusion**	High, low or undetectable	Visible	Variable	Variable
**Total occlusion**	Undetectable	Visible, no detectable lumen	Not applicable	Not applicable

Adapted from Grant EG, Benson CB, Moneta GL, Alexandrov AV, Baker JD, Bluth EI, et al. Carotid artery stenosis: gray-scale and Doppler US diagnosis--Society of Radiologists in Ultrasound Consensus Conference. Radiology 2003;229:340-346, Copyright ©2003, with permission from the Radiological Society of North America.

S2 Table: Criteria for the diagnosis of intracranial stenosis (using recommendations from Tsivgoulis et al, and Zhao et al) [2, 3]

<table>
<tr><th rowspan="2">Artery</th><th rowspan="2">Depth</th><th rowspan="2">Stenosis grade</th><th colspan="2">Primary criteria</th><th colspan="2">Secondary criteria</th><th rowspan="2">Other useful parameters</th></tr>
<tr><th>Difference with contralateral side</th><th>MFV</th><th>SPR</th><th>PSV</th></tr>
<tr><td rowspan="4">MCA</td><td rowspan="4">30 – 65</td><td>Normal</td><td>< 30%</td><td>< 80</td><td>< 2</td><td>< 120</td><td rowspan="16">- Direction of the flow (signal above or below the midline)
- Aspect of the Doppler signal (turbulence, blunted or not)
- Sound of the Doppler signal (murmurs, vibrations)
- Confounders: age, fever, anaemia</td></tr>
<tr><td>< 50%</td><td>≥ 30%</td><td>80 - 100</td><td>< 2</td><td>120 - 140</td></tr>
<tr><td>50 – 69%</td><td>≥ 30%</td><td>100 – 120</td><td>2 – 3</td><td rowspan="2">> 140</td></tr>
<tr><td>≥ 70%</td><td>≥ 30%</td><td>> 120</td><td>≥ 3</td></tr>
<tr><td rowspan="4">ICA siphon</td><td rowspan="4">60 – 65</td><td>Normal</td><td>< 30%</td><td>< 80</td><td>< 2</td><td rowspan="2">< 120</td></tr>
<tr><td>< 50%</td><td>≥ 30%</td><td>80 - 100</td><td>< 2</td></tr>
<tr><td>50 – 69%</td><td>≥ 30%</td><td>80 – 100</td><td>2 – 3</td><td rowspan="2">> 120</td></tr>
<tr><td>≥ 70%</td><td>≥ 30%</td><td>> 100</td><td>NA</td></tr>
<tr><td rowspan="3">PCA†</td><td rowspan="3">55 – 70</td><td>Normal</td><td>< 30%</td><td>< 60</td><td>< 2</td><td>< 100</td></tr>
<tr><td>50 – 69%</td><td>≥ 30%</td><td>60 - 100</td><td>2 – 3</td><td rowspan="2">> 100</td></tr>
<tr><td>≥ 70%</td><td>≥ 30%</td><td>> 100</td><td>≥ 3</td></tr>
<tr><td>ACA</td><td>60 – 75</td><td colspan="5">Same criteria as for MCA</td></tr>
<tr><td>BA</td><td>70 – 100+</td><td colspan="5" rowspan="2">Same criteria as for PCA</td></tr>
<tr><td>VA</td><td>45 – 70</td></tr>
</table>

MCA = Middle Cerebral Artery, ICA = Internal Carotid Artery, PCA = Posterior Cerebral Artery, ACA = Anterior Cerebral Artery, BA = Basilar Artery, VA = Vertebral Artery (this refers only to the intracranial portion).

† The PCA has a small and highly variable diameter. Asymmetry between the right and the left is frequently reported due to anatomical variations. Therefore, there is no reliable ultrasound criteria to define a <50% PCA stenosis. Such diagnosis is usually made via computed tomography/magnetic resonance/digital subtraction angiography).

References

1.Grant EG, Benson CB, Moneta GL, Alexandrov AV, Baker JD, Bluth EI, et al. Carotid artery stenosis: gray-scale and Doppler US diagnosis--Society of Radiologists in Ultrasound Consensus Conference. Radiology 2003;229:340-346.
2.Tsivgoulis G, Neumyer MM, Alexandrov AV. Diagnostic criteria for cerebrovascular ultrasound. In: Alexandrov AV, editor. Cerebrovascular Ultrasound in Stroke Prevention and Treatment. 2nd ed. Sussex: Wiley-Blackwell; 2011. p. 87-143.
3.Zhao L, Barlinn K, Sharma VK, Tsivgoulis G, Cava LF, Vasdekis SN, et al. Velocity criteria for intracranial stenosis revisited: an international multicenter study of transcranial Doppler and digital subtraction angiography. Stroke 2011;42:3429-3434.
